# Caring for People With Multiple Chronic Conditions

**DOI:** 10.5888/pcd12.150438

**Published:** 2015-11-12

**Authors:** Susan M. Smith

The systematic review by Bleich and colleagues contributes to the much needed evidence supporting management of patients with multiple chronic conditions — multimorbidity. Although the review’s focus is the United States, it addresses an issue that is highly relevant to all health care systems and raises important questions about how we can design systems to address the needs of this vulnerable group (1). Literature is now well-established on the impact of multimorbidity on patients, health care providers, and health care systems (2,3), but less is known about interventions that can improve outcomes in this group of patients (4).

The systematic review of Bleich et al provides evidence from 27 studies that examined 5 models of care and suggests that care management, case management, and disease management are promising models for people with multimorbidity. This finding seems to fit with our intuitive sense that care coordination will reduce the challenges experienced by people with multiple chronic conditions, related polypharmacy, and high use of health care services (5). However, evidence from other settings does not yet support a move to widespread implementation of case management for multimorbidity, and many questions remain regarding the optimal design of such programs (6). In addition, we need to avoid imposing an additional treatment burden on a population that is already struggling with potential over-exposure to medical care (7).

The review of Bleich et al also highlights the heterogeneity within the population of people with multiple chronic conditions and their overlap with other patient groups in relation to disability and frailty. Definitions of multimorbidity and related constructs such as comorbidity continue to be debated (8), but the definition of multimorbidity as 2 or more conditions can be regarded as the norm now, for older people in particular (9). Current evidence suggests that we need to focus on the subgroup of patients with multiple conditions who can be regarded as having complex multimorbidity on the basis of a high number of conditions, polypharmacy, and high health care use (10). These definitional and operational aspects of multimorbidity highlight the importance of clearly reporting patient characteristics and settings when evaluating interventions for these patients. Reporting is essential to allow consideration of external validity or generalizability of multimorbidity interventions for those considering introducing interventions in other settings (11).

An additional key issue that Bleich et al discuss is the need for appropriate comparison or control groups to allow for robust evaluation. Ideally, randomized trials would be employed that use cluster and stepped wedge designs where appropriate. Only 13 of the 27 studies included in this review were randomized controlled trials (RCTS), which may partially explain the differences between Bleich’s review and the related Cochrane review. The Cochrane review included only RCTs and not less robust study designs, and the included studies were from a wider range of countries, including the United States (4). Variations in outcomes reported across different studies are also challenging, and work is ongoing to develop a core outcome set for multimorbidity research.

The systematic review of Bleich et al highlights the need for further evidence to support policy and management of patients with complex multimorbidity. In the meantime, studies should be designed to allow robust evaluation of interventions and should build on the evidence from this and related reviews that suggest a health care focus on patient-centered outcomes for patients with multimorbidity.
